# Cancer of the Breast

**Published:** 1904-11

**Authors:** 


### CANCER OF THE BREAST.

The subject of carcinoma of the breast was discussed at the Southern Surgical and Gynecological Association by Dr. W. F. Westmoreland, Professor of Surgery in the Atlanta College of Physicians and Surgeons. He declared that carcinoma now presents even a more serious problem than formerly, on account of its rapid increase. The proportionate mortality from cancer is four and a half times greater now than half a century ago. No other disease can show anything like such an immense increase. According to statistics, two out of every five cases of carcinoma in the female are of the breast. Over three-fourths of all the tumors occurring in the breast are carcinoma, or to be exact, of 638 tumors, 530 or 83.20 per cent. are carcinoma, leaving 107 or 16.79 per cent. representing all other forms of neoplasms. Speaking of the pathology, he said there was no subject in surgery about which such divergent opinions are held. There is a wide variation in the malignancy of carcinoma, some proving fatal in less than a year, a few others taking ten or fifteen years. He gave a classification of carcinoma into simply the scirrhous and medullary or encephaloid. The former has an average duration of life of about thirty months, the latter of about twelve months. When it is considered that the skin and pectoral muscles are involved in nearly every instance in this disease, the surgeon can easily see the philosophic reasoning upon which Halstead's operation is based, which Westmoreland prefers. He believes, however, that Dr. Bloodgood was the first to demonstrate the advantages of completely cleaning out the posterointernal subscapular region by the supraclavicular route. The results of the Halstead operation, in the opinion of the doctor, depend upon the ability of the individual operator, his earliness in seeing the case and his closeness in following Halstead's technic. This operation not only requires surgical skill and experience, but proper respect for the magnitude of small things. In the opinion of Dr. Westmoreland, no surgeon will make a satisfactory operation on his first few cases. He finds that with each case he operates more satisfactorily. The operation does not present the seriousness that its magnitude would lead us to believe. He has operated six times before his class at a public clinic and transferred the patients afterward, at times quite a distance, to their homes. In his first operations he had some shock from exposure, but this he easily controlled by placing hot towels over the exposed surfaces. There is practically no blood lost in a properly-made operation. It is better to complete the operation, skin-grafting included, at one sitting. When, for any reason, this is not practicable, the cleaning out of the supraclavicular region and the grafting can be made at a second operation. When grafting is done at a second operation, he finds it better to remove the granulations. This is best done by curetting. In two of his cases it was necessary to remove the subscapular nerves, and in another to resect the axillary vein. No unpleasant sequence followed in either case. He has operated upon over fifty cases of carcinoma since he began surgical work, and has always cleaned out the axilla and stripped the muscles in each case. He does not know the results in the majority of these cases. Two of them, however, came back to him with later recurrence, one with a carcinoma of the femur five years after the operation, no local return; the other with a local return in the cicatrix nine years afterward. This case died in a short time from metastasis. He feels quite sure that these were not independent new growths. There is always one rule that the more malignant the case, the greater the blood-changes.—*American Journal of Surgery and Gynecology.*

